# Revisiting El-Sayed Synthesis: Bayesian Optimization for Revealing New Insights during the Growth of Gold Nanorods

**DOI:** 10.1021/acs.chemmater.4c00271

**Published:** 2024-02-27

**Authors:** Anish Rao, Marek Grzelczak

**Affiliations:** † Centro de Física de Materiales CSIC-UPV/EHU, Paseo Manuel de Lardizabal 5, 20018 Donostia San-Sebastián, Spain; ‡ Donostia International Physics Center (DIPC), Paseo Manuel de Lardizabal 4, 20018 Donostia-San Sebastián, Spain

## Abstract

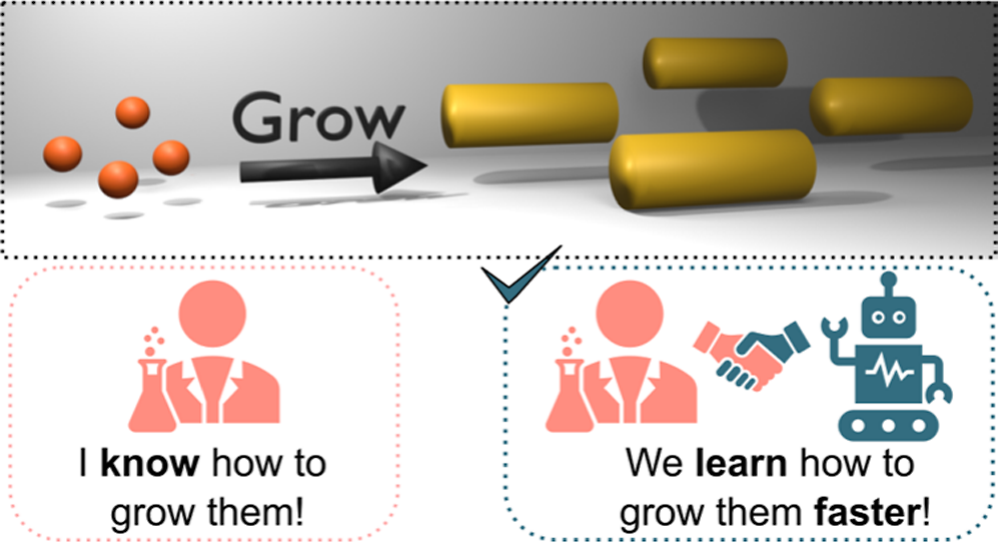

In diverse fields, machine learning (ML) has sparked transformative changes, primarily driven by the wealth of big data. However, an alternative approach seeks to mine insights from “*precious data*”, offering the possibility to reveal missed knowledge and escape potential knowledge traps. In this context, Bayesian optimization (BO) protocols have emerged as crucial tools for optimizing the synthesis and discovery of a broad spectrum of compounds including nanoparticles. In our work, we aimed to go beyond the commonly explored experimental conditions and showcase a workflow capable of unearthing fresh insights, even in well-studied research domains. The growth of AuNRs is a nonequilibrium process that remains poorly understood despite the presence of well-established seeded growth protocols. Traditional research aimed at understanding the mechanism of AuNR growth has primarily relied on altering one reaction condition at a time. While these studies are undeniably valuable, they often fail to capture the synergies between different reaction conditions, thus constraining the depth of insights they can offer. In the present study, we exploit BO, to identify diverse experimental conditions yielding AuNRs with similar spectroscopic characteristics. Notably, we identify viable and accelerated synthesis conditions involving elevated temperatures (36–40 °C) as well as high ascorbic acid concentrations. More importantly, we note that ascorbic acid and temperature can modulate each other’s undesirable influences on the growth of AuNRs. Finally, by harnessing the power of interpretable ML algorithms, complemented by our deep chemical understanding, we revisited the established hierarchical relationships among reaction conditions that impact the El-Sayed-based growth of AuNRs.

## Introduction

In recent years, machine learning (ML)-based methods have been successfully applied to a wide variety of problems like improving the spectroscopic features of data,^1,2^ finding information from data too complex to analyze,^3^ designing and predicting structures,^4^ new materials,^5,6^ reactions,^7^ etc. Much of this progress is attributed to the advent of “*big data*”, which empowers complex analysis and interpolations. Big data-driven research endeavors often result in the prediction of materials with novel and often unprecedented properties. An alternate strategy exploits and harnesses the advances made in statistics to gain insights not necessarily from “*big data*”, but from “*precious data*”.^8^ Such research pursuits are paramount for revealing “*missed knowledge*” and escaping “*knowledge traps*” that both researchers and ultimately ML methods are prone to.^8−11^ In this direction, among the array of techniques, black-box optimization protocols, such as Bayesian optimization (BO), have emerged as invaluable tools for optimizing complex functions through surrogate models and utility functions.^12^ BO-based approaches have significantly contributed to the design, discovery, and optimization of organic reactions,^13^ development of materials for organic light-emitting diodes,^14^ refinement of formulations,^15^ synthesis of materials^16,17^ and nanoparticles (NPs),^18,19^ etc. Within this diverse landscape, the optimization of gold nanoparticles (AuNPs) enjoys a compelling pursuit due to their multifaceted applications^20^ spanning medicine,^21^ biotechnology,^22^ and catalysis.^23,24^ Of particular interest is the optimization of anisotropic AuNPs like Au nanorods (AuNRs) that have superior plasmonic, photothermal, and catalytic properties than other NP geometries.^25^ While previous works have been carried out for the automated optimization of AuNRs (putting human out of the loop),^26,27^ our approach diverges and finds more fundamental aspects by posing: Can BO-based protocols be employed to uncover “*new knowledge*” within the domain of AuNR growth? We define “*new knowledge*” as one that ventures into unexplored chemical space, revealing hitherto unknown synergies between reaction conditions.

In the domain of AuNR synthesis, numerous well-established protocols have been developed, notably those pioneered by the groups of El-Sayed,^28^ Murphy,^29^ Murray,^30^ Liz-Marzán,^31,32^ and others.^33,34^ Additionally, considerable research has been devoted to understanding the mechanism governing the synthesis of AuNRs using a diverse array of techniques such as small-angle X-ray scattering,^35,36^ high-end electron microscopy,^37,38^ simulations,^39−41^ controlled synthesis conditions,^42,43^ design of experiments,^44^ etc. Most often, such studies rely on one-factor-at-a-time experimentation to study the influence of reaction parameters on AuNR growth. Moreover, the parameter space explored in all of these studies is rather small, as well. While these studies have provided valuable insights,^45−50^ they often fail to capture the intricate synergies between different reaction conditions, thereby limiting the depth of understanding they impart.^44^ Although studies utilizing design of experiments (DoE) are essential, BO offers several advantages, including efficient optimizations and the ability to handle categorical variables, leveraging complex predictive models.^51^ This makes BO particularly attractive for material synthesis. Despite the valuable insights generated by DoE studies, our knowledge is limited as only a small portion of the parameter space has been explored for the growth of AuNRs. Moreover, a detailed analysis of different reaction conditions leading to viable growth of AuNRs has not been conducted to date. Furthermore, the growth of AuNRs is a nonequilibrium process governed by a chemical reaction network featuring nonlinear relationships that remain poorly understood.^52^ Given this complexity, it becomes essential to pinpoint the minimal set of reactants capable of growing AuNRs. This is crucial for achieving a deeper understanding of the entire growth process. With this in mind, we plan to use a BO-based workflow to reveal potential synergies and relationships among the conditions governing the growth of AuNRs. More specifically, we wanted to study whether BO-based strategies can reveal new knowledge on two important facets. First, what diverse experimental conditions can yield AuNRs with similar spectroscopic characteristics? Second, by harnessing the power of ML, can we identify the reaction conditions that are critical for the growth of AuNRs?

In the present work, we aim to showcase a two-step strategy combining BO and ML to reveal missed insights in the domain of AuNR growth. By optimizing and interpreting various AuNR growth conditions, we aim to enhance our comprehension of the synthesis process for AuNRs. Here, Gryffin,^53^ a BO-based experimental planner renowned for its effectiveness in AuNP synthesis,^54^ plays a pivotal role in orchestrating an unbiased exploration of the parameter space. We varied five experimental conditions, namely, amounts of silver (Ag^+^), ascorbic acid (A.A.), hydrochloric acid (HCl), seeds and the temperature of the reaction to identify distinct experimental conditions that yield AuNRs. We note that BO-based techniques can find previously unknown reaction conditions that produce AuNRs having similar spectroscopic properties within a relatively low number of experiments. Intriguingly, we observed the viable synthesis of AuNRs having localized surface plasmon resonance (LSPR) in the NIR region (∼800 nm) that can be carried out at elevated temperatures, as high as 39 °C. More specifically, we demonstrate the presence of conditions, where the growth of AuNRs can be carried out ∼4.7 times faster than standard experimental conditions, without compromising on their spectroscopic properties. In addition to finding alternate and arguably better experimental conditions for the production of AuNRs, we found synergies between two experimental conditions that govern the growth of AuNRs, i.e., the temperature of the reaction and A.A. Temperature is known to accelerate the production of AuNRs, but it inevitably results in reducing their aspect ratios and ultimately reducing the LSPR maxima of AuNRs.^55,56^ Furthermore, higher concentrations of A.A. are known to induce secondary nucleation events^57^ and the growth of irregular structures.^58^ Intriguingly, we note that A.A. and temperature can balance each other’s undesirable effects, i.e., secondary nucleation^57,58^ and decreasing aspect ratios of AuNRs,^55,56^ respectively. This capacity of parameters to possibly counteract each other’s undesirable influences constitutes a novel contribution to the existing literature. Furthermore, the observation of alternate and broader reaction conditions for the growth of AuNRs challenges the prevalent belief that AuNRs with comparable spectroscopic properties can only be grown in a narrow range of reaction conditions.^32,52,59^ Finally, we employed a random forest regressor, complemented by our deep chemical understanding,^60^ to analyze the data set compiled during the course of our study, for establishing a hierarchy of reaction conditions that impact the growth of AuNRs. Collectively, the broadening of AuNR growth conditions, revealing synergies between A.A. and temperature and establishing the importance of reaction conditions, enhance our understanding of the processes governing AuNR growth.

## Experimental Section

### Synthesis of AuNRs

AuNRs were synthesized in accordance with the well-established seeded-growth synthesis method.^28^

#### Au Seeds

Au seeds (∼1.5 nm) were prepared by the fast reduction of HAuCl_4_ (5 mL, 0.25 mM) with freshly prepared NaBH_4_ (0.3 mL, 10 mM) in aqueous cetyltrimethylammonium bromide (CTAB) solution (100 mM) under vigorous stirring for 2 min at room temperature and were then kept undisturbed at 27 °C for 30 min to ensure the complete decomposition of sodium borohydride. The mixture changed from light yellow to brownish, indicating the formation of Au seeds. Note that the UV–vis–NIR characteristics of the prepared seeds were measured to confirm the complete reduction of Au^+^.

#### AuNR Growth

In a typical experiment, to a 10 mL solution of 100 mM CTAB, 100 μL of HAuCl_4_ (50 mM) was added, and the system was stirred for 5 min to ensure completion of the Au(III)–CTAB complex. Next, 104 μL of Ag^+^ (10 mM), 480 μL of A.A. (100 mM), and 207 μL of HCl (1 M) were sequentially added to the solution. Within a few seconds after the addition of A.A., the solution turned from yellow to colorless. Finally, 32 μL of Au seeds was added to the solution, and the system was left undisturbed for 2 h at 34 °C. Here, the amounts of A.A., Ag^+^, HCl, and Au seeds and the temperature of the reaction were obtained using Gryffin. At the end of 2 h, the UV–vis–NIR characteristics of the unpurified AuNRs were measured to calculate the loss.

### UV–Vis–NIR Studies

Extinction spectra of as-prepared AuNRs were recorded in the spectrophotometer in a polystyrene cuvette (10 mm path length) in the 400–1100 nm wavelength range. Kinetic experiments (shown in Figure 2) were performed in a quartz cuvette with a path length of 1 mm. All of the analysis and plotting were performed using Python. The raw data and code can be accessed on the GitHub repository at https://github.com/anishrao/Pr-AuNR-opt. During the course of the optimization campaign, each experiment was performed only once. However, after the optimized reaction conditions were identified, the experiment was replicated three more times.

## Results and Discussion

### Optimizing the Synthesis of AuNRs

**Figure 1:**
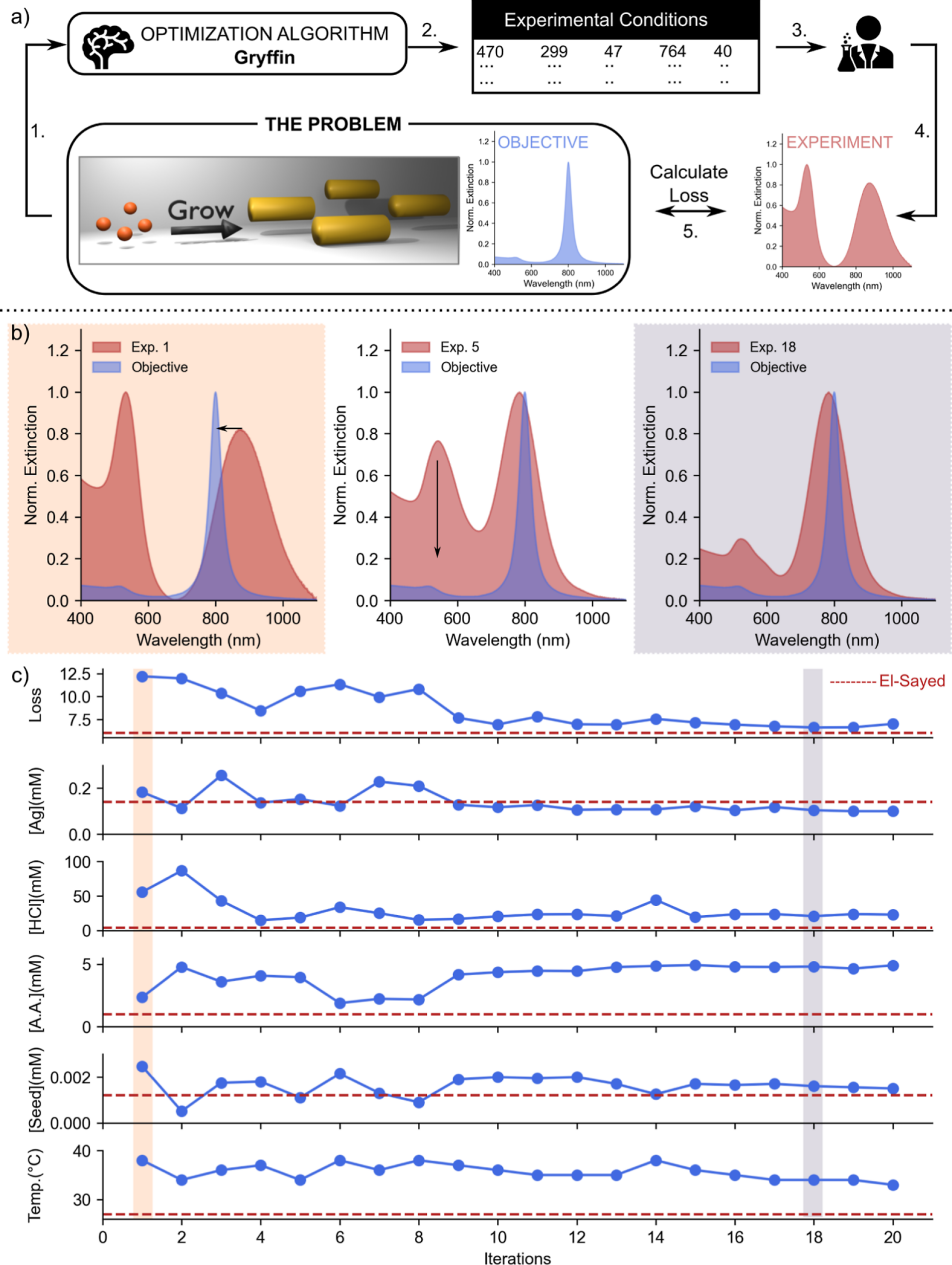
(a) Schematic illustrating the workflow utilized for the optimization of AuNRs using El-Sayed’s protocol. Here, the calculated spectrum of one single AuNR with the LSPR band at 800 nm was given to Gryffin for optimization. (b) Representative UV–vis–NIR spectra obtained at the beginning (shaded in orange), and at the end (shaded in blue) of an optimization experiment. (c) Variations in different experimental conditions and the loss values during the complete optimization experiment.

A unique characteristic of the present investigation is to see if planning algorithms can be exploited to reveal new information that can be “*added*” to the existing knowledge pool. Our specific aim was to investigate whether diverse experimental conditions could lead to the formation of AuNRs with comparable spectroscopic properties. This pursuit was driven by the desire to discover and understand the synergistic interactions among various experimental parameters governing the synthesis of AuNRs. With this in mind, we selected the synthesis of AuNRs using the well-known El-Sayed protocol since it contains a small number of chemicals and does not use additives^28^ as our test problem (see Experimental Section for synthesis details). The present work uses Atinary,^61^ a web application allowing the optimization of experiments. We used Gryffin, an experimental planning algorithm, to optimize the synthesis of AuNRs. To begin with, we provided the calculated spectrum of a single AuNR (length: 56 nm; width: 16 nm) as the objective spectrum (blue spectrum in Figure 1a). This approach mitigates potential limitations of experimentally obtained UV–vis–NIR spectra, which can vary between researchers and be influenced by the synthesis quality. Offering a calculated spectrum serves as the universal ground truth and, therefore, a more dependable objective. The task for Gryffin was to propose iteratively the reaction conditions for the growth of AuNRs with minimal spectroscopic differences with this objective spectrum. The complete workflow is shown in Figures 1a and S1. In El-Sayed’s protocol, five key parameters, namely, amounts of Ag^+^, A.A., HCl, and seeds and temperature of the reaction can be varied to tune the spectroscopic properties of AuNRs.^42^ These reaction conditions, accompanied by the parameter boundaries mentioned in Table 1 were introduced as continuous variables within Gryffin’s framework. Subsequently, the optimization process proceeded with experiments performed by a researcher (human-in-the-loop; step 3, Figure 1a) under conditions obtained using Gryffin (step 2, Figure 1a). Here, having human-in-the-loop is suitable to circumvent the complexities associated with creating an automated NP synthesis platform—a nontrivial task.^62,63^ This nontriviality stems from a range of reasons like high investment of cost and time in setting up autonomous liquid handling platforms, challenges associated with replicating actions that are easy for humans due to their fine motor skills and hand–eye coordination, etc.^62,63^ Finally, following an incubation and growth period of 2 h, the UV–vis–NIR spectrum of the resultant AuNRs was measured (step 4, Figure 1a). In order to inform the algorithm how similar the objective and experimentally obtained UV–vis–NIR spectra are, we defined a parameter loss. Here, loss is defined as the L2-norm of the difference between the normalized UV–vis–NIR spectrum of as-prepared and objective AuNRs (see Section S2 for more details). We used the spectrum of as-prepared AuNRs so that the presence of impurities can act as the “cost” of the synthesis conditions. In this context, impurities refer to the formation of isotropic byproducts such as spheres or cubes during the growth of AuNRs.^59^ The existence of these isotropic shapes will result in increased absorbance in the 400–600 nm region, ultimately contributing to high loss values—a representation of a costly synthesis. Minimizing this loss value results in both matching the LSPR band as well as minimizing the byproducts formed during the growth process. Here, employing the calculated value of loss as the objective simplifies the typically complex task of optimizing NP synthesis, a task that frequently demands multiobjective optimization.^54^ The value of loss was then passed onto Gryffin to guide the optimization process, constituting step 5 in Figure 1a. Iteratively, the identification of effective recipes for the synthesis of AuNRs was performed in a maximum of 20 experiments.

**Table 1:** Table Showing the Boundary Limits for the Parameters Given to Gryffin

	A.A. (μL)	silver (μL)	HCl (μL)	seeds (μL)	temp (°C)
upper bound	100	100	100	10	25
lower bound	500	400	1000	50	40

The five-dimensional parameter space involving 41,895 possible experiments (see Section S3 in the Supporting Information) was navigated using Gryffin as the experiment planner. Figure 1b shows the representative variations in the UV–vis–NIR spectra of AuNRs at selected experimental iterations. Here, the blue spectrum shows the objective spectrum provided to the algorithm, and the red spectrum shows the obtained UV–vis–NIR spectrum of as-prepared AuNRs. We can clearly note that within 5 iterations, the algorithm found a way to match the longitudinal LSPR band to minimize the loss, and in the subsequent iterations it learned to minimize the production of byproducts during the synthesis. Figure 1c shows the variations in the loss from 12.5 (indicating the formation of impure AuNRs) to 6.33 (indicating the formation of pure good-quality AuNRs) during the 20 iterations of experiments. The variations in the UV–vis–NIR spectrum during the course of the experiment are shown in Figure S2. Here, one can clearly see the formation of impure AuNRs with a mismatched LSPR band and slowly the matching of LSPR peaks, along with the decreasing formation of impurities within around 12 iterations. More importantly, we observed a viable growth of AuNRs under experimental conditions involving high temperature and a high amount of A.A. that are distinctly different from the ones used for AuNR synthesis using standard conditions.^28^ In Figure 1c, the dashed red lines denote the experimental conditions commonly used during El-Sayed synthesis. Specifically, we observed viable AuNR synthesis at 4.35, 1.33, 3.4, and 6× higher concentration of A.A., high temperature, and high amounts of HCl and seeds, respectively. Commonly, it is assumed that the growth of AuNRs with desired LSPR bands can occur under a narrow range of experimental conditions.^32,59^ Our work reveals that a broad range of reaction conditions exist that give rise to AuNRs with similar spectroscopic properties (Figure 1c). It is worth mentioning that we are strictly assessing the spectroscopic features of AuNRs using the calculated values of loss. More specifically, the similar the value of loss, the more similar the AuNRs are.

**Figure 2:**
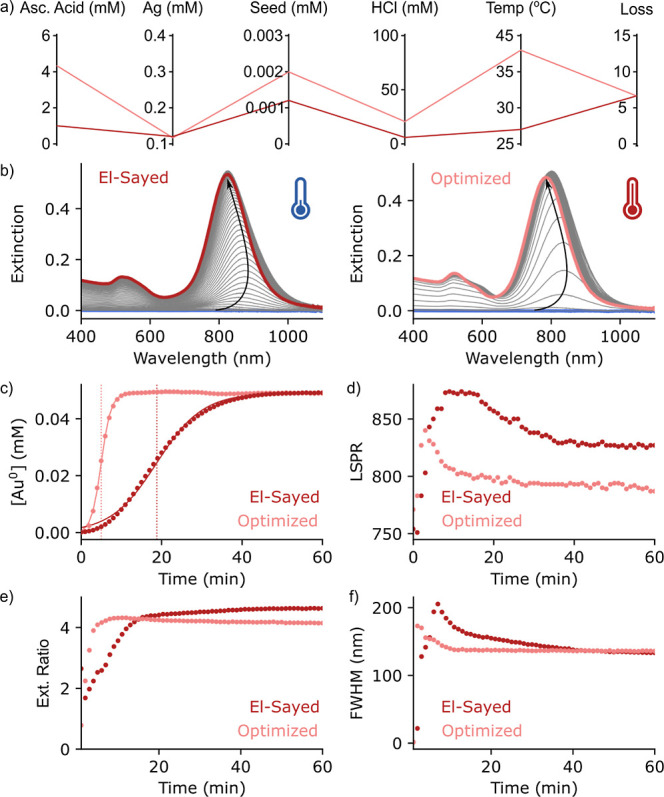
(a) Parallel coordinate graph showing different experimental conditions that resulted in the growth of AuNRs with similar spectroscopic features. (b) Time-dependent UV–vis–NIR variation during the growth of AuNRs under standard (left) and optimized (right) experimental conditions. Variation in the (c) conc. of Au^0^ and (d) LSPR with time, showing the rate of growth of AuNRs under standard and optimized experimental conditions. Variation of (d) Ext. at LSPR/Ext. at 400 nm, and (e) full-width at half maxima (fwhm) of the LSPR peak, plotted as a function of time, showing the spectroscopic similarity between both AuNR samples.

We observed that distinctly different temperatures, seed amounts, A.A. and HCl concentrations can yield AuNRs with comparable spectroscopic qualities (Figure 2a). Notably, AuNRs with LSPR maxima near 800 nm can be synthesized even at 36 °C, in contrast to 27 °C during the conventional El-Sayed protocol. The parallel coordinate graph shown in Figure 2a clearly illustrates the contrasting experimental conditions employed in our optimized and El-Sayed AuNR growth protocols. This observation carries significant implications since high-temperature AuNR synthesis typically reduces the aspect ratios of AuNRs, ultimately leading to a blue shift in their LSPR maxima.^55,56^ While high-temperature synthesis of AuNRs presents challenges for achieving high aspect ratios, it remains an enticing endeavor due to the accelerated growth kinetics.

Notably, previous research exploring temperature effects on AuNR growth has often fallen short in elucidating the intricate interdependencies and synergies between parameters. Specifically, can alternative parameter combinations enable the accelerated production of AuNRs without compromising their aspect ratios? To comprehensively explore the synthesis conditions, we conducted a kinetic study under both El-Sayed and optimized conditions. Figure 2b,c shows the time-dependent variations in the UV–vis–NIR spectrum during the growth of AuNRs under El-Sayed and newly optimized conditions, respectively. In Figure 2c, we studied the variations in concentration of Au^0^ during the synthesis of AuNRs. We can note that the synthesis of AuNRs with analogous LSPR bands occurs on vastly different time scales, with standard synthesis requiring at least 50 min and the new optimized conditions achieving completion in just 10 min. It is important to note that although the reduction occurs at different time scales, we observe ∼100% reduction of Au^3+^ to Au^0^ under both reaction conditions. More importantly, the rate of reduction of Au^0^ at 36 °C is 4.69 times the rate obtained at 27 °C. This highlights a key achievement of our study—an accelerated production of high-quality AuNRs with similar spectroscopic signatures.

Next, we investigated the temporal evolution of the LSPR peak position under the two growth conditions (Figure 2d). In both scenarios, the longitudinal LSPR displayed the typical red shift for the first few minutes of reduction, followed by a gradual blue shift until the reduction of the Au precursor was complete.^64^ Importantly, the extent of blue shifts was more pronounced for the optimized conditions (∼57 nm) compared to standard El-Sayed conditions (∼47 nm) due to increasing temperature.^64^ The similarity between AuNRs synthesized using El-Sayed’s and our newly optimized reaction conditions was established by comparing the extinction ratio at the LSPR peak, 400 nm (Figure 2e), and the full-width at half-maxima (fwhm, Figure 2f) of grown AuNRs. Here, the extinction ratio was used to assess the presence of impurities,^59^ while the fwhm of the LSPR peak was used to assess the polydispersity of grown AuNRs. In Figure 2e, we observe a marginally higher extinction ratio for AuNRs grown using El-Sayed conditions (∼4.5) over optimized ones (∼4.1). This is possibly due to the slightly higher aspect ratio of AuNRs obtained using El-Sayed conditions, as indicated by the higher LSPR peak position (827 nm) compared to the 783 nm obtained during optimized growth conditions (see Figure 2d). It is well known that with an increasing aspect ratio of AuNRs, the extinction ratio also increases.^59,64^ Furthermore, we observed an increase in the fwhm, followed by a significant blue shift of 74 nm for El-Sayed conditions, as opposed to a shift of 37 nm in our optimized reaction conditions. This observation aligns with the known effect of temperature on fwhm during the growth of AuNRs.^64^ More importantly, we observe that the fwhm of AuNRs obtained using both experimental conditions is similar (∼131–135 nm). Collectively, these comparisons establish the validity of loss as an optimal metric to capture the multiobjective features in the UV–vis–NIR spectrum of AuNRs. Finally, we studied the similarities between the two AuNRs using transmission electron microscopy (TEM) measurements. The lengths and widths of optimized AuNRs are 48.9 ± 9.2, and 19.9 ± 4.4 nm, respectively. While these measurements appear similar to the dimensions of our target AuNR (length = 56 nm and width = 16 nm), it is important to note that a precise size comparison necessitates optimization experiments explicitly designed with dimensions as the primary objective. Nonetheless, akin to the UV–vis–NIR comparisons between AuNRs obtained under El-Sayed and optimized conditions, Figure S3 revealed similar sizes and size-distribution histograms for the AuNRs synthesized under these different conditions. The marginal differences in lengths, widths, and ultimately aspect ratios of AuNRs grown under both experimental conditions are reflected in their size distributions as well (Figure S3). Specifically, the length (57.0 ± 6.3 nm) is greater and the width (16.7 ± 2.7 nm) is smaller for AuNRs produced through El-Sayed synthesis than those achieved through optimized synthesis, resulting in a higher aspect ratio of AuNRs grown using El-Sayed’s protocol. This trend is consistent with results obtained using UV–vis–NIR analysis, where El-Sayed synthesis yields AuNRs with larger aspect ratios compared to the optimized synthesis. These comprehensive UV–vis–NIR and TEM analyses highlight the similarity in UV–vis–NIR response, yields, and sizes of AuNRs synthesized under both El-Sayed and optimized conditions in the present study.

### Reproducibility and Flexibility

**Figure 3:**
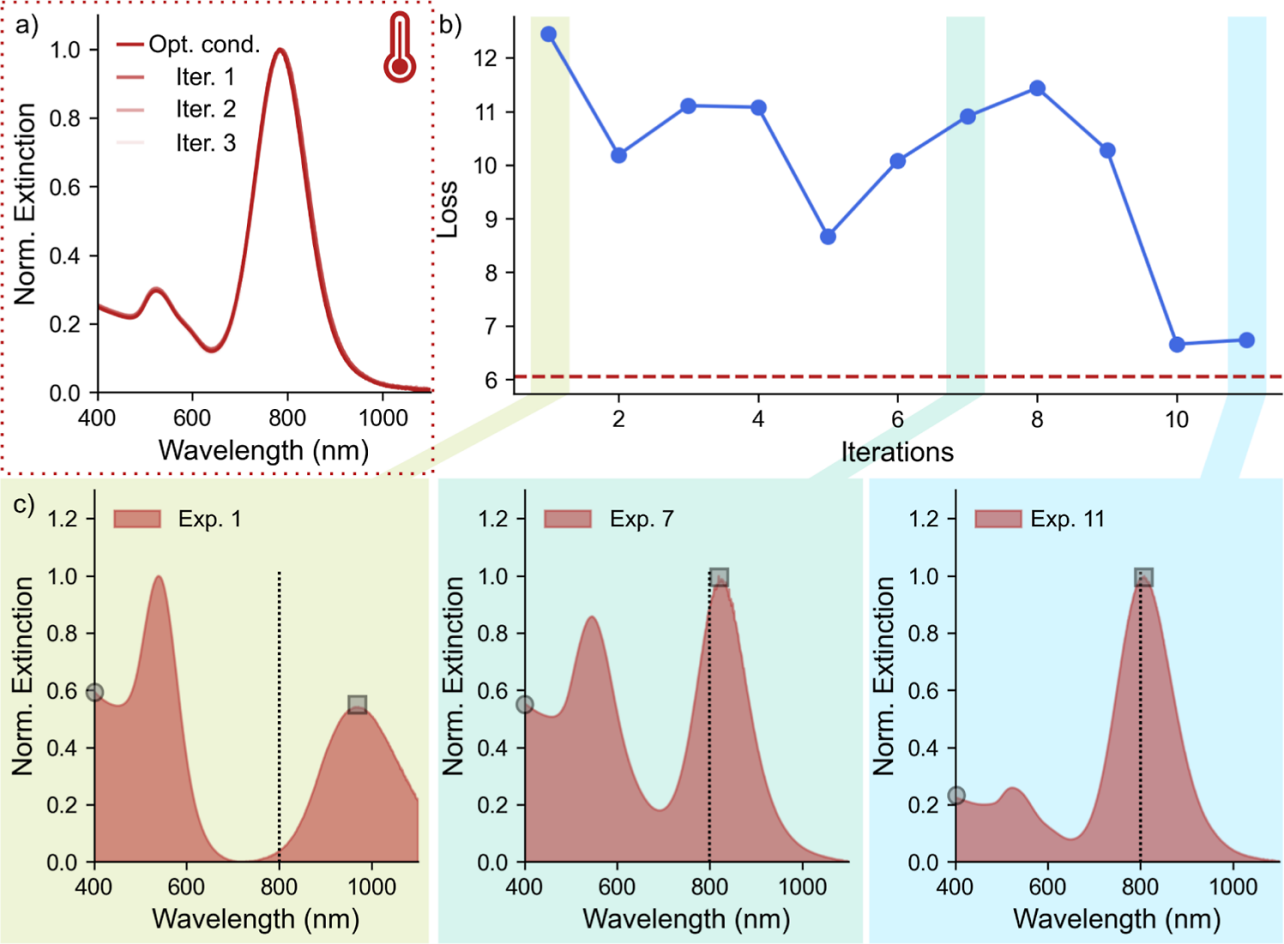
(a) UV–vis–NIR spectrum of three AuNRs synthesized under optimized reaction conditions. (b) Variations in the loss of AuNRs with a multiobjective optimization experiment. (c) Representative UV–vis–NIR spectra obtained during the progress of the optimization experiment. Here, the LSPR band matching (shown by a dotted line) and the ratio between the extinction values shown by the square and circle were used as the two objectives.

The next critical question to be addressed is the reliability and reproducibility of these “*new*” experimental conditions for AuNR synthesis. With this in mind, we performed and reproduced the AuNR growth process under the newly optimized conditions three more times (see parts 1–3 in Figure 3a). Importantly, for all three experimental trials, we obtained AuNRs with similar loss values (ranging between 6.58 and 6.72) and, ultimately, similar UV–vis–NIR spectra (see Figure 3a). This underscores that any experimental errors, though existent, are minimal and unlikely to substantially impact the key takeaways of our study. Moreover, to comprehensively explore the parameter space, we sought to investigate the possibility of repeating the entire optimization process from various initial points within the parameter space. With this in mind, we instructed Gryffin to initiate the optimization experiment from a different point, representing alternative initial reaction conditions. These reaction conditions were autonomously chosen using Gryffin and showed no resemblance to established literature protocols. This is evident not only from the varying amounts of reagents used in these syntheses (see the dataset provided on github repository, and Table S1 in the Supporting Information) but also from the growth of undesirable AuNRs under these reaction conditions. With this in mind, we initiated the optimization experiment from a different point serving as the initial reaction condition. Intriguingly, within 9 iterations, we successfully synthesized high-quality AuNRs (see Figure S4). Notably, this synthesis too occurred at elevated temperatures and A.A. concentrations, specifically at 39 °C and 4.52 mM, respectively. Interestingly, even after performing multiple optimization experiments, we observed a consistent growth of AuNRs at conditions involving high amounts of A.A. and high temperatures (see Figure S5). This observation reveals that A.A. and temperature can potentially balance each other’s undesirable effects. It is crucial to acknowledge that the optimization of synthesis is not always guaranteed. Throughout various iterations stemming from diverse initial reaction conditions in the parameter space, there were instances in which pure AuNRs were not achieved within 20 iterations. The resulting AuNR dispersion exhibited substantial absorbance across the 400–600 nm range, indicating the presence of isotropic byproducts (see Figure S6). It is plausible that by configuring the algorithm with a greater inclination for exploration, coupled with a higher number of iterations, it might converge toward the synthesis of high-quality AuNRs from these starting points as well. However, our primary objective was to explore diverse AuNR growth recipes rather than assert the inevitability of AuNR formation from all conceivable experimental conditions.

Next, we investigated the algorithm’s adaptability in altering the objective of optimization. In this context, instead of minimizing spectral disparities with calculated AuNRs, we prioritized target properties such as the LSPR wavelength (as denoted by the black dotted lines in Figure 3c) and the ratio of extinction at the LSPR (represented by squares) to extinction at 400 nm (represented by circles) in Figure 3c. Gryffin allows the flexibility of performing multiobjective optimizations by accommodating the definition of multiple desired target properties using Chimera,^65^ a hierarchical scalarizing function. Notably, by giving higher precedence to maximizing the absorbance ratio, thus ensuring the formation of AuNRs with minimal byproducts, we achieved successful AuNR optimization within 11 iterations (Figures 3 and S7). The resulting AuNRs exhibited a loss value of approximately 6.66, signifying the production of AuNRs of similar quality under these altered optimization criteria, as well.

**Figure 4:**
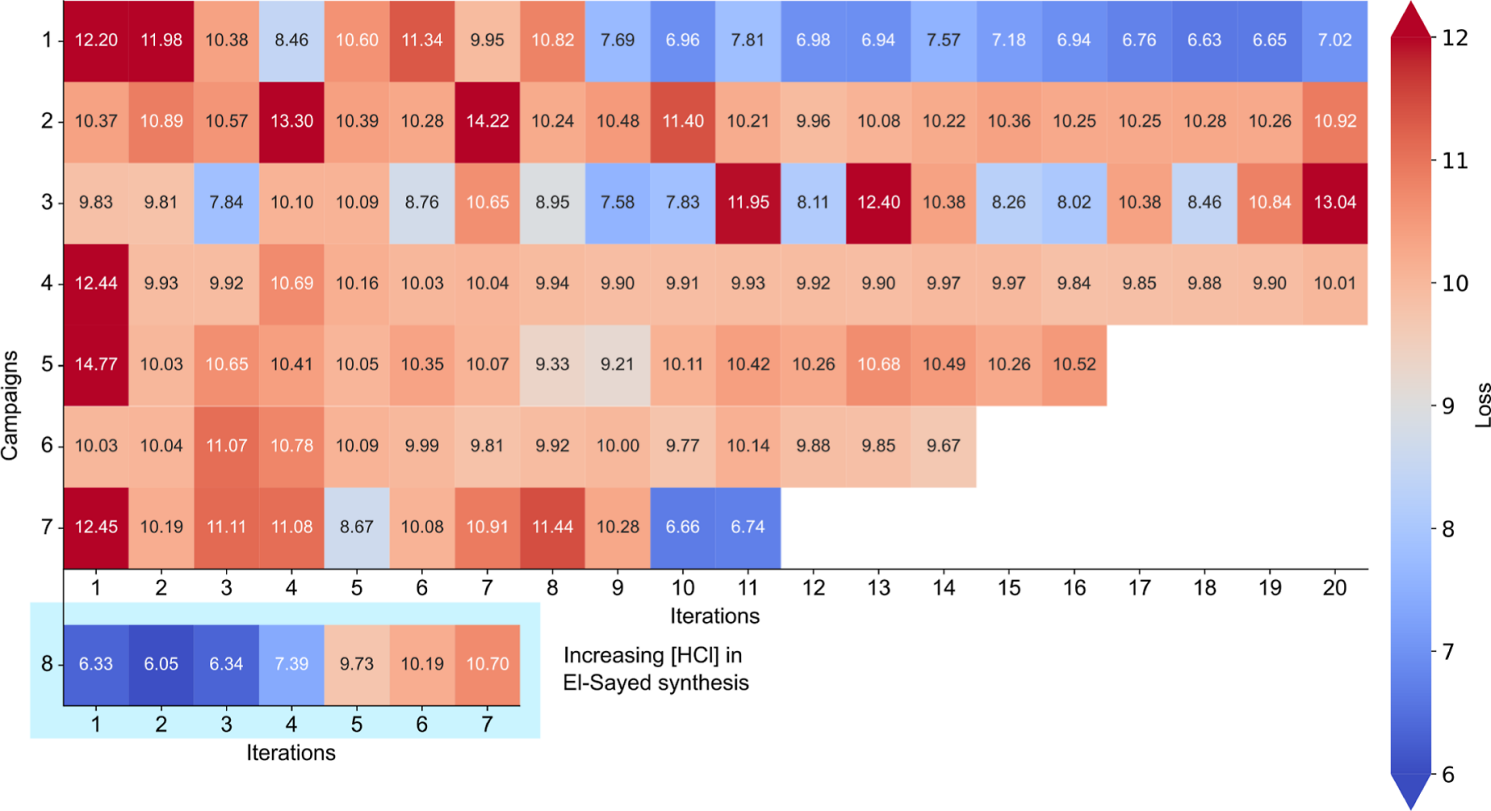
Heatmap of the data set that was generated during the course of the study. The *x*-axis illustrates the outcomes of AuNR growth across various experiments within a single optimization campaign, while the *y*-axis corresponds to results obtained from different optimization campaigns, each originating from a distinctly different point within the parameter space. Here, the blue tiles denote the formation of good AuNRs, i.e., AuNRs with loss values less than 8.

The primary advantage of performing experiments based on BO lies in the generation of a *good data set*. Here, a good data set refers to data that is personalized, as opposed to relying on data sets sourced from various external laboratories. The data set comprises 121 optimization experiments and encompasses experimental conditions relevant to the production of both high-spectroscopic features, as well as impure AuNRs.^66^Figure 4 shows the heatmap of the data set that was generated during the course of the study. The *x*-axis illustrates the outcomes of AuNR growth across various experiments within a single optimization campaign, while the *y*-axis corresponds to results obtained from different optimization campaigns, each originating from a distinctly different point within the parameter space. Figure 4, therefore, encapsulates the diversity of AuNR growth outcomes under different experimental conditions within the parameter space. The color and numerical value of each tile convey the similarity of the resulting AuNRs in the parameter space to the target UV–vis–NIR spectrum. Tiles in blue signify acceptable AuNRs, while those in red indicate unacceptable outcomes. Campaign8 in Figure 4 illustrates the variations in loss values resulting from experiments performed under diverse HCl concentrations (i.e., effect of pH) while following the conditions outlined in the El-Sayed protocol. Finally, we trained an ML model on our personalized data set, aiming to unravel the intricate relationships between reaction conditions and their impact on the overall AuNR growth process.

### Hierarchy of Parameters during AuNR Synthesis

Now, we delve into a deeper analysis of the dataset featured in the prior section. To unlock the hidden insights within this data set, we harnessed the capabilities of PyCaret, an open-source library in Python that automates ML workflows.^67^ Our objective was to discern whether the variations in the reaction conditions could serve as reliable predictors of the loss value associated with the resultant AuNRs. The data set consists of five features, i.e., five experimental conditions and one label, i.e., loss value. Although our data set contains a lower proportion (∼15%) of experiments at conditions giving loss values less than 8, they hide a wealth of information that can be extracted using suitable ML models.^66^ Our rigorous analysis revealed that among the array of models examined, the random forest regressor emerged as the top performer in forecasting the outcomes of loss values. The training data set exhibited an *R*^2^ value of 0.946, highlighting reasonably good predictive accuracy. Moreover, even on unseen testing data, the model demonstrated robust performance with an *R*^2^ value of 0.711 (see Figure S8). For more details on the ML model, see Sections S10 and S12 in the Supporting Information.

Next, we conducted an in-depth analysis to determine the relative significance of each experimental parameter on the spectroscopic properties, specifically the loss values, of the synthesized AuNRs. We employed the framework of SHAP to understand the impact of different reagents and reaction conditions on the spectroscopic features of AuNRs (Figure S9). Here, the reaction conditions are arranged from top to bottom in a descending order, reflecting their decreasing influence on the predicted loss values.

According to this analysis, Ag^+^ emerged as the most prominent contributor to achieving AuNRs with small loss values. This observation aligns with the pivotal role of silver in orchestrating the symmetry-breaking event,^37,68^ a critical step in the growth of AuNRs. Surprisingly, the concentration of HCl during synthesis emerged as the second most influential factor, surpassing the importance of A.A. It is known that A.A. primarily serves as the reducing agent responsible for converting Au^3+^ to Au^1+^. Furthermore, elevated levels of A.A. are known to induce secondary nucleation events^57^ and the growth of irregular structures.^58^ While the primary role of HCl is to modulate the reduction potential of A.A., ultimately tuning the aspect ratio of AuNRs.^42^ Intuitively, therefore, one would have ascribed more importance to A.A. over HCl. This deviation between the experimental intuition and the importance ascribed by the model is possibly due to the way loss is calculated. It should be noted that both the presence of byproducts and the mismatch between the experimental and calculated LSPR bands result in increasing loss values. Figure S10 clearly shows the formation of AuNRs (with low byproducts) but with increasing LSPR mismatch. The algorithm interprets this tuning of LSPR as the production of impure AuNRs, and erroneously ascribes it higher importance than A.A. Further, temperature and the quantity of seeds exhibited comparatively weaker effects on the synthesized AuNRs. Seed quantity predominantly governs the volume of the resultant AuNRs, with higher seed amounts yielding smaller volume AuNRs.^32,69^ It is worth noting that even temperature has a relatively mild influence on the preparation of AuNRs centered at 800 nm. The wide spectrum of experimental conditions capable of yielding comparable quality AuNRs may stem from the relatively modest impact of factors such as A.A. and reaction temperature. This limited influence likely creates numerous alternative conditions suitable for the synthesis of AuNRs.

**Figure 5:**
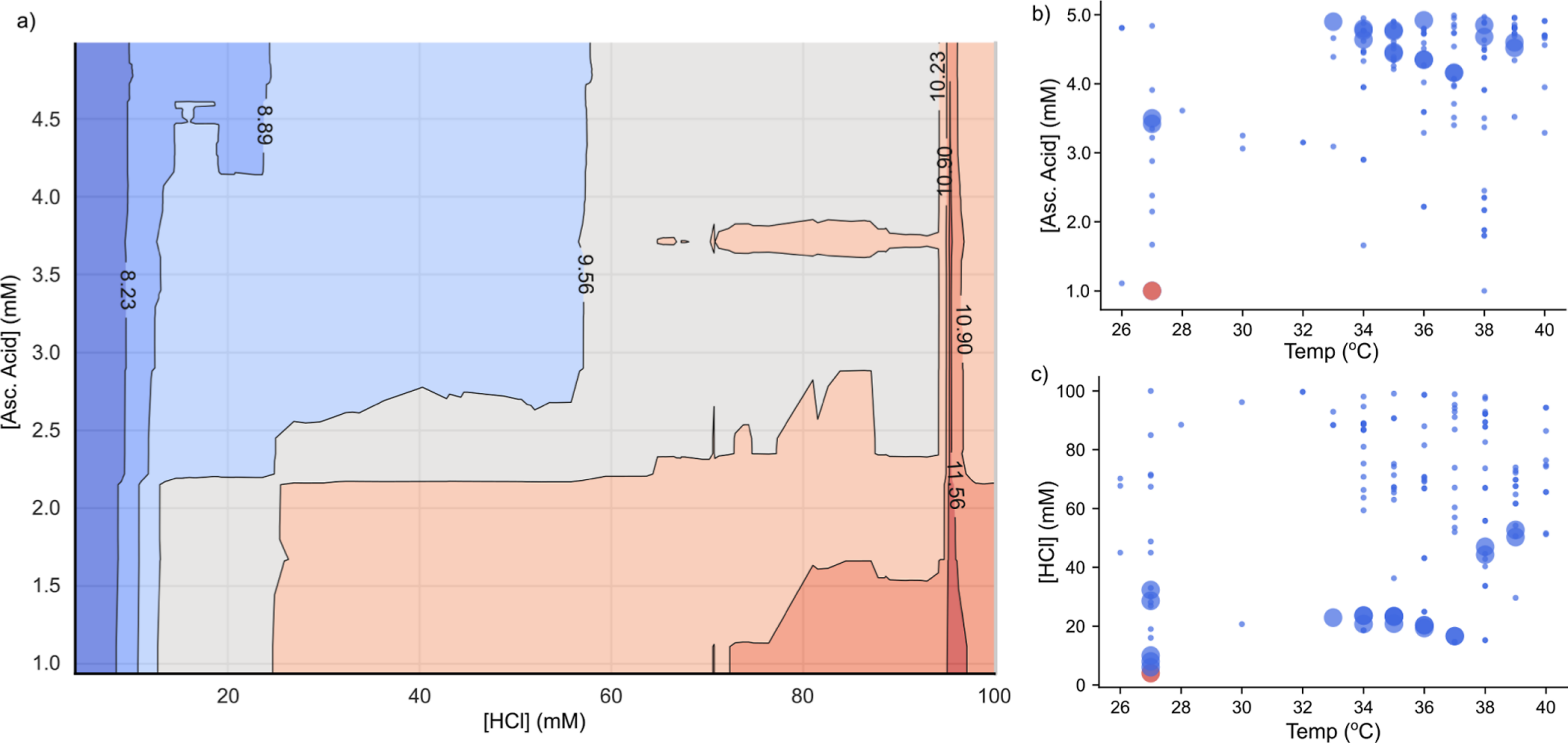
(a) Partial dependence plot showing the collective effect of A.A. and HCl on the loss values predicted using the trained ML model. Scatter plot showing the effect of (b) A.A./temperature and (c) HCl/temperature on the experimentally obtained loss values of grown AuNRs. Here, points with large sizes indicate the successful formation of AuNRs with low loss values (i.e., loss less than 8). The pink circles denote the conditions used during El-Sayed synthesis.

While the SHAP analysis provides valuable insights, it lacks the ability to capture the synergistic interactions among multiple reaction conditions that influence the growth of AuNRs. To address this limitation and gain a comprehensive understanding, Figures 5 and S11 demonstrate the collective effect of two distinct reaction conditions on loss values using partial dependence plots. While the partial dependence plots may not explicitly illustrate the capacity of one reaction parameter to counteract the adverse effects of another, they do demonstrate how a combination of multiple reaction conditions can be employed to grow AuNRs with comparable UV–vis–NIR spectra. Figure S11 illustrates that among the range of tested experimental conditions, Ag^+^ offers the least flexibility (indicated by a small blue region), whereas Au seeds show the highest flexibility (indicated by a large blue region). Figure 5a presents the partial dependence plots for A.A. and HCl, two important reaction conditions identified using SHAP analysis. Figure 5a reveals that a wide range of A.A. and HCl concentrations can be used to synthesize AuNRs with similar spectroscopic features. Note that the high loss values (shown by red contours) with increasing HCl concentration is attributed to the red shifts in the LSPR peaks rather than the presence of impurities during the AuNR growth process (Figure S10). In addition to revealing the impact of selected features on the predictions made by the trained ML model, Figure 5b,c illustrates the relationships between A.A., HCl, temperature, and experimentally obtained loss values. Large circles highlight experiments that produce high-quality AuNRs, i.e., experiments with loss values below 8. Consistent with the insights from interpreting the ML model, Figure S12 reveals that except for Ag^+^, most experimental conditions offer considerable flexibility in concentrations to produce AuNRs with similar spectroscopic features. An important finding from this investigation is the demonstration that a wide variety of experimental conditions can yield AuNRs with similar spectroscopic features. These conditions, although result in the growth of similar AuNRs, follow distinct pathways. For instance, as highlighted in the section on optimizing the synthesis of AuNRs, one of the newly discovered synthesis conditions demonstrated accelerated AuNR production, promising large-scale adoption across various industries. There are several key takeaways from the present study. First, we demonstrate the growth of AuNRs with similar UV–vis–NIR responses in a diverse range of experimental conditions and not only in a small section of the parameter space. Second, we establish a hierarchy of experimental parameters that govern the growth of AuNRs. Third, we show the potential ability of temperate to modulate the undesired secondary nucleation or growth of truncated structures associated with high AA.

## Conclusions

Our work significantly deviates from the conventional workflows that employ ML-based methods to predict new materials or BO-based protocols to optimize the formation of a desired material. In the present work, we propose and demonstrate a workflow where BO can be exploited for revealing “*missed knowledge*” during the growth of AuNRs. We harnessed BO, specifically Gryffin, to systematically explore diverse experimental conditions that yield AuNRs with similar spectroscopic features. We could successfully demonstrate the existence of reaction conditions in which synergies between experimental conditions can be exploited to yield similar AuNRs through different pathways. Specifically, we show the viable synthesis of AuNRs in an uncharted territory (i.e., high A.A. concentration and high temperature) that goes against the human-based or intuition-guided conventional knowledge for growing NPs.^45^ Additionally, our innovative approach enabled the discovery of previously unrecognized synergies between A.A. and temperature, wherein they can modulate each other’s undesirable influences on the growth of AuNRs. Finally, the accelerated production of high-quality AuNRs serves as a promising avenue for the accelerated as well as large-scale production of AuNRs. The implications of our findings extend far beyond the AuNR synthesis. This study exemplifies the transformative potential of BO and ML in escaping “*knowledge traps*” and revealing missing knowledge. While the insights garnered in this study are significant, this represents our preliminary investigation in our overarching objective of harnessing BO, interpretable ML, and high-throughput experimentation for generating new chemical knowledge. In the future, our goal is to integrate the current workflow with high-throughput experimentation and extend our exploration to a wider parameter space. This endeavor aims to establish and comprehend the design principles governing the growth of AuNPs of various shapes and sizes.

## Data Availability

All the data and code are available at https://github.com/anishrao/Pr-AuNR-opt.

## Supporting Information Available

Methodology for loss calculation; schematic demonstrating the experimental workflow; UV–vis–NIR and TEM measurements for AuNR growth under different experimental conditions; diverse experimental conditions for successful AuNR growth; and residual, SHAP, and partial dependence plots using ML (PDF)
