# Supplementary material for: Revisiting El-Sayed Synthesis: Bayesian Optimization for Revealing New Insights during the Growth of Gold Nanorods
Source: Chem Mater. 2024 Feb 27;36(5):2577–87. doi: 10.1021/acs.chemmater.4c00271 (PMC11049742; doi:10.1021/acs.chemmater.4c00271)
Supplement: Supplementary file 1 — cm4c00271_si_001.pdf [file cm4c00271_si_001.pdf]

Supporting Information for Revisiting El-Sayed Synthesis: Bayesian Optimization for Revealing New Insights During Growth of Gold Nanorods

Anish Rao*,† and Marek Grzelczak*,†,‡

†Centro de Física de Materiales CSIC-UPV/EHU, Paseo Manuel de Lardizabal 5, 20018 Donostia San-Sebastián, Spain

‡Donostia International Physics Center (DIPC), Paseo Manuel de Lardizabal 4, 20018 Donostia-San Sebastián, Spain

E-mail: anish.rao@ehu.eus; marek.g@csic.es

S1 Materials and Methods

S1.1 Chemicals

Hexadecyltrimethylammonium bromide (CTAB for molecular biology, ≥99%), hydrogen tetrachloroaurate trihydrate ($HAuCl_4 \cdot 3\,H_2O$, ≥99.9%), silver nitrate ($AgNO_3$, ≥99.0%), L-ascorbic acid (A.A., ≥99%), and sodium borohydride ($NaBH_4$, 99%), Hydrochloric acid (HCl, 37%) were purchased from Sigma-Aldrich and used without further purification. Milli-Q grade water (resistivity 18.2 MΩ cm at 25°C) was used in all experiments. All glassware were cleaned with aqua regia, rinsed with Milli-Q water, and dried before use.

S1.2 Data and Code

All data and the code used to obtain the figures shown in the main text and the supporting information is available at https://github.com/anishrao/Pr-AuNR-opt. The code was written in Jupyter Notebook and the environment used during the course of the study is also provided in the project folder. The dataset produced during the course of the study is shared as a separate supporting information file (Dataset-all-data.csv).

We used PyCaret (version 3.1.0) to train and compare twenty machine-learning models on our dataset. All models were evaluated using a ten-fold cross-validation protocol. For training the model, 70 % of the data was used to train the model, while the performance of the models was tested on 30% of the remaining data.

S1.3 Transmission Electron Microscopy (TEM) Studies

We performed TEM experiments in order to characterize the dimesnsions of AuNRs formed. Samples for TEM were prepared using 200 μL of as-prepared AuNRs (in 100 mM CTAB) that was centrifuged and redispersed in 200 μL of Milli-Q water. This purification step was repeated twice, and after the last centrifugation cycle, the AuNRs were dispersed in 20 μL of H_2O. Finally, 5 μL of drop was placed on formvar coated Copper grid. The drop was allowed to slowly evaporate overnight at room temperature before imaging.

S1.4 Modeling of Optical Properties

Simulations of optical spectra were conducted using the boundary element method (BEM).[1,2] For simulations, we chose AuNR (in water) with a length of 56 nm and a width of 16 nm. The dielectric data for Au were taken from Johnson and Christy.[3]

S2 Calculate Loss

One of the challenges in the optimization of NPs originates from the necessity to target multiple spectroscopic properties (like peak position, reduction of Au^0, presence of impurities, full-width half maxima, etc.) at the same time. In the present study, we define a parameter *loss* that combines all targeted into a single merit. We calculate the value of loss in the following way:-

- Normalize the objective ($UV_{calc.}$) and experimental ($UV_{exp.}$) UV-Vis-NIR spectra.
- Calculate the difference between the two spectra i.e. $\Delta UV = UV_{exp.} - UV_{calc.}$.
- Calculate the L-2 norm of ΔUV

The L-2 norm of a vector is defined as follows:-

$$L_2(\Delta_{UV}) = \sqrt{{x_1}^2 + {x_2}^2 + {x_3}^2 + ... + {x_{700}}^2} \tag{1}$$

S3 Calculate the Experimental Parameter Space

The limits allowed for different reaction conditions are mentioned in Table 1. In order to get a rough estimate of the total number of possible experiments that need to be performed, we performed the following calculation. For instance, imagine A.A., Ag^+ and HCl can be screened in discrete increments of 50μL. The seeds are screened in discrete amounts of 10μL, and temperature in increments of 2.5°C. This discretization gives us a choice of 9 A.A., 7 Ag^+, 5 seed, 19 HCl and 7 temperature values. These values give rise to **41,895** possible combinations of experiments that need to be performed. It should be noted that the more resolved the search space, the higher the number of experiments that need to be performed. The discretization followed in the above estimate gives a lower bound to the possible number of experiments that need to be performed.

S4 Schematic Showing the Experimental Workflow

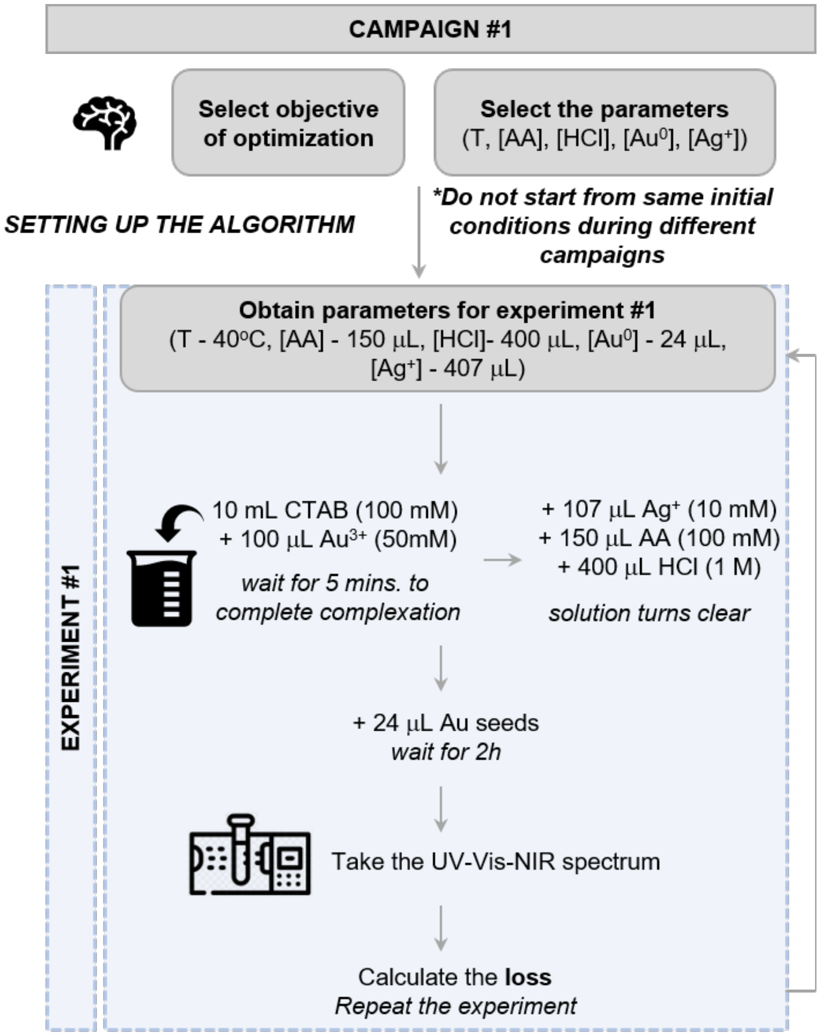

Figure S1: Schematic showing the complete experimental workflow. Here, optimizing the growth of AuNRs with minimal spectroscopic differences to the calculated UV-Vis-NIR spectra was chosen as the objective. Next, 5 experimental conditions were varied in a continuous fashion, to perform the optimization experiments.

S5 UV-Vis-NIR for all Experiments in Figure 1

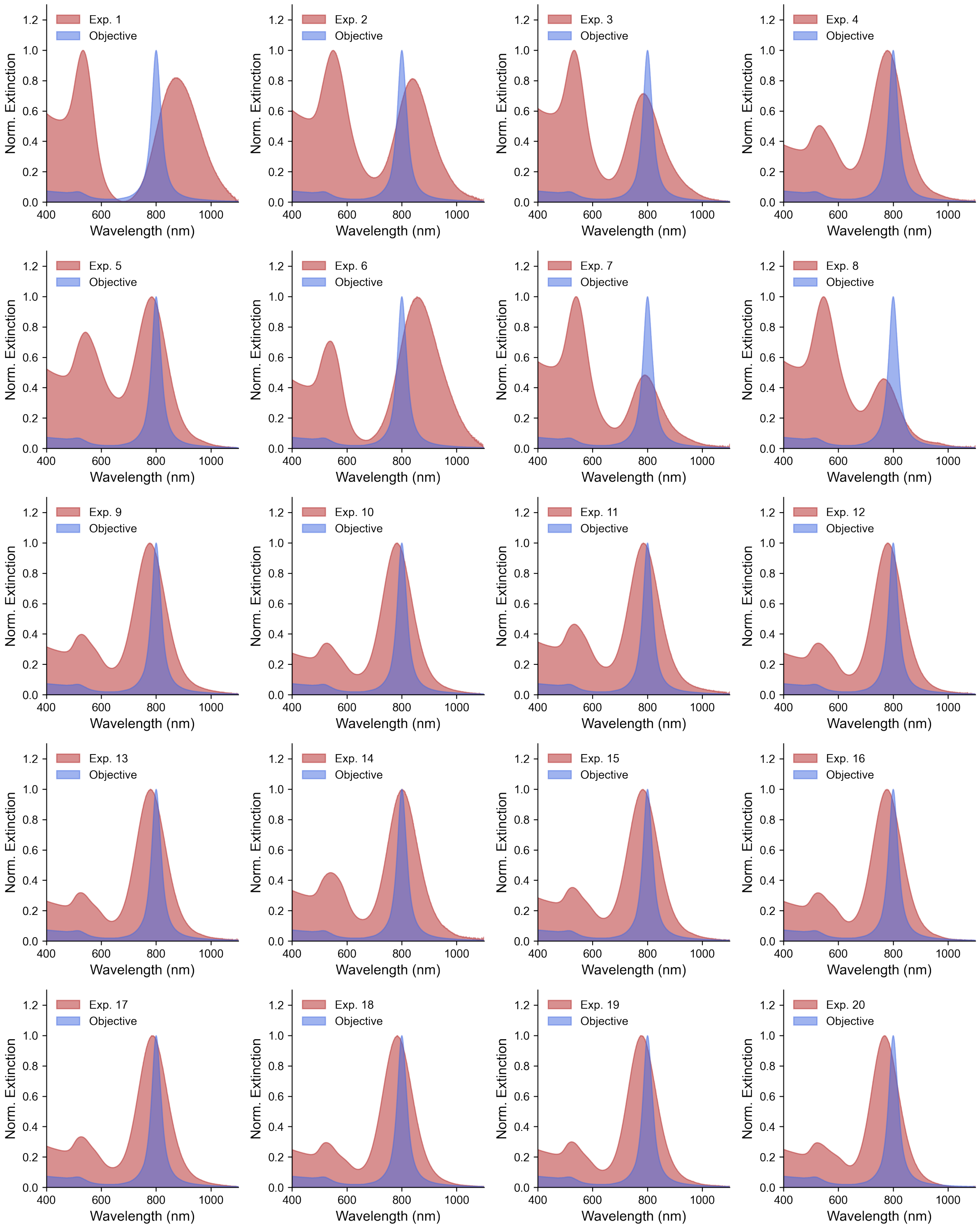

Figure S2: UV-Vis-NIR spectrum of all the experiments performed during the first iteration towards optimization of AuNRs.

S6 TEM Characterization of Optimized AuNRs

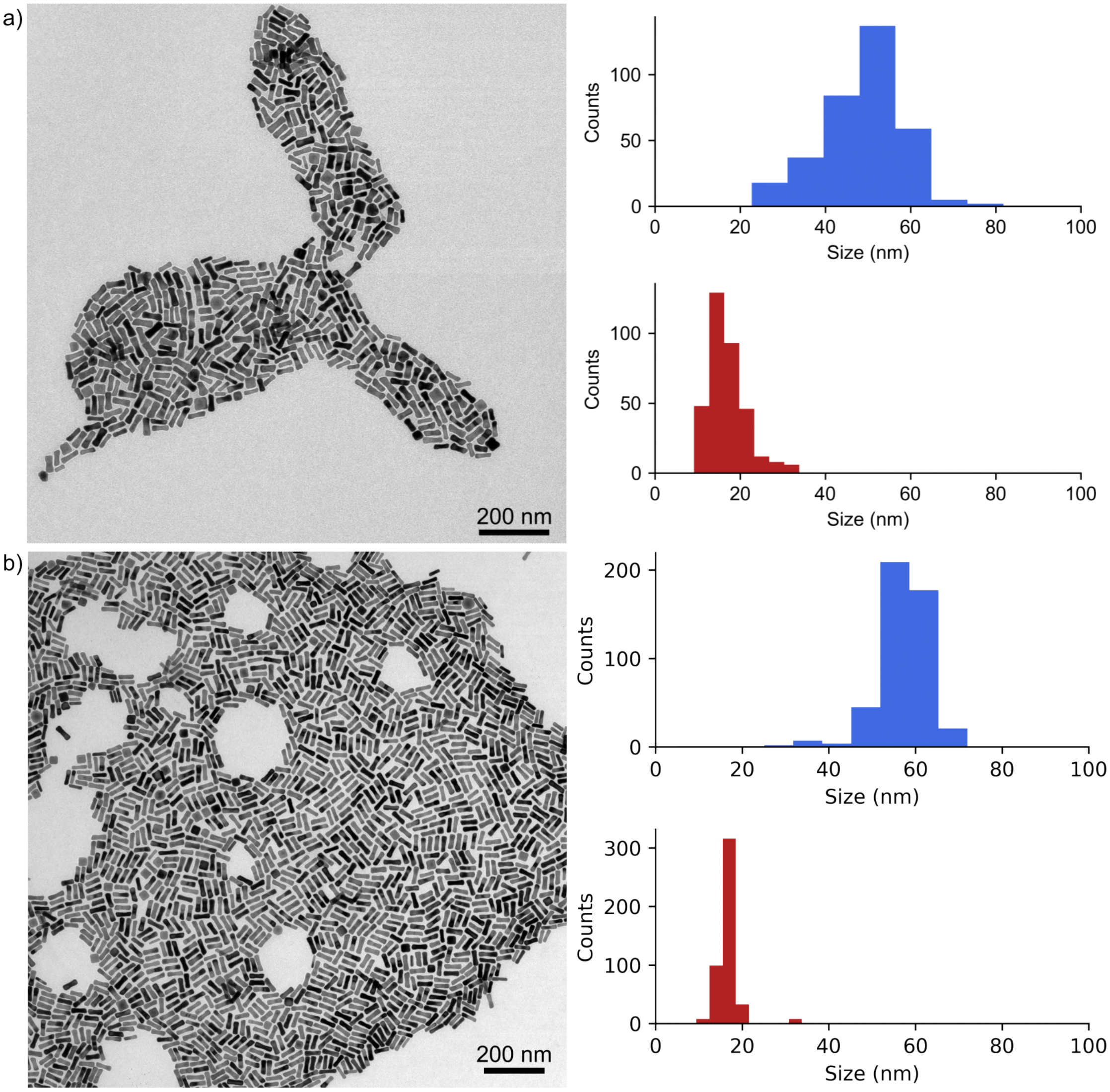

Figure S3: TEM image and accompanying size distribution histogram showing the length (shown in blue) and width (shown in red) of AuNRs synthesized using a) optimized and b) El-Sayed conditions. The length and width of optimized AuNRs are 48.9 ± 9.2, and 19.9 ± 4.4 nm respectively, while the length and width AuNRs grown under El-Sayed conitions are 57.0 ± 6.3, and 16.7 ± 2.7 nm respectively.

S7 UV-Vis-NIR Variations from Different Initial Points

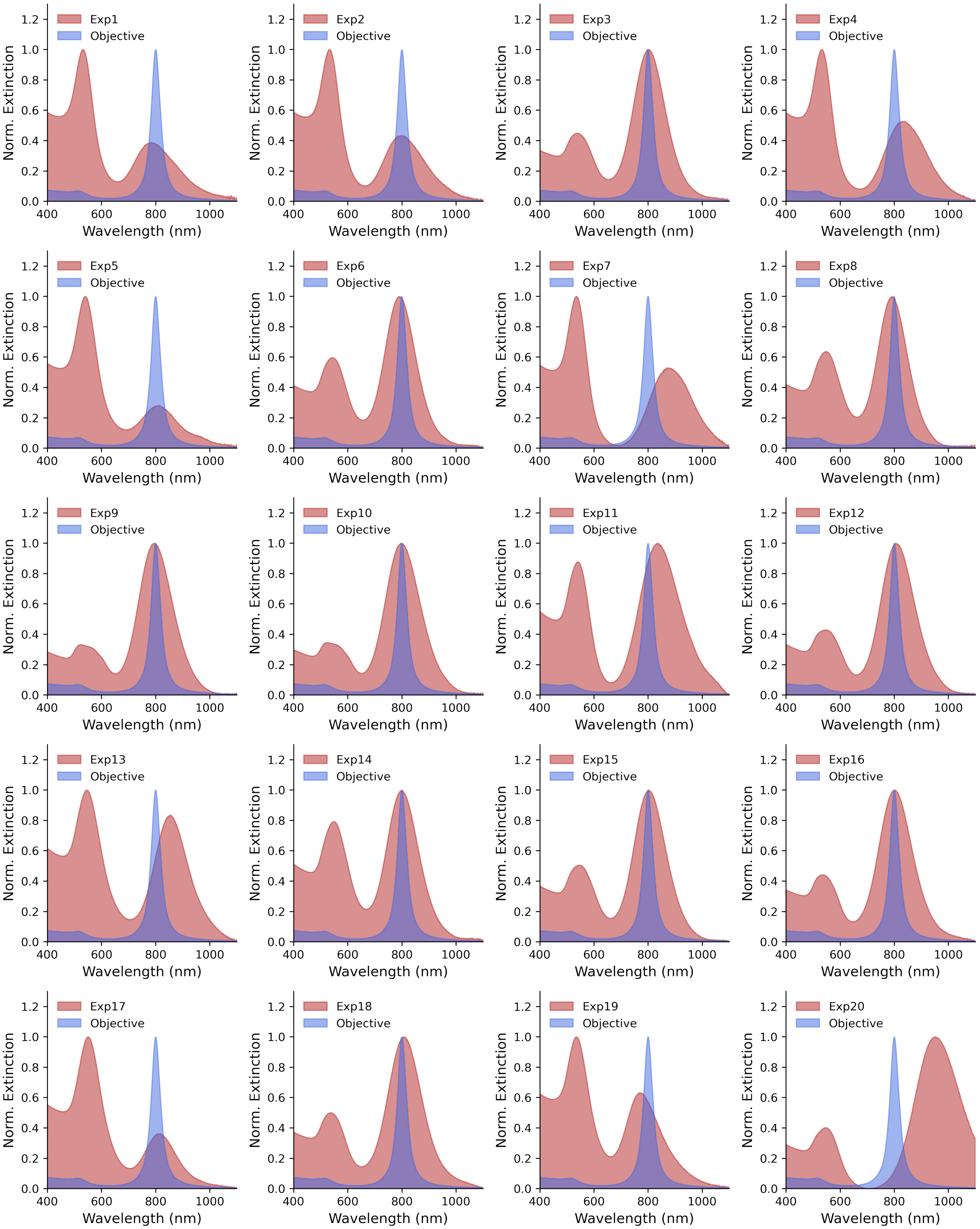

Figure S4: UV-Vis-NIR spectrum of all the experiments performed during the successful iteration towards optimization of AuNRs.

S8 Diverse Reaction Conditions for AuNR Growth

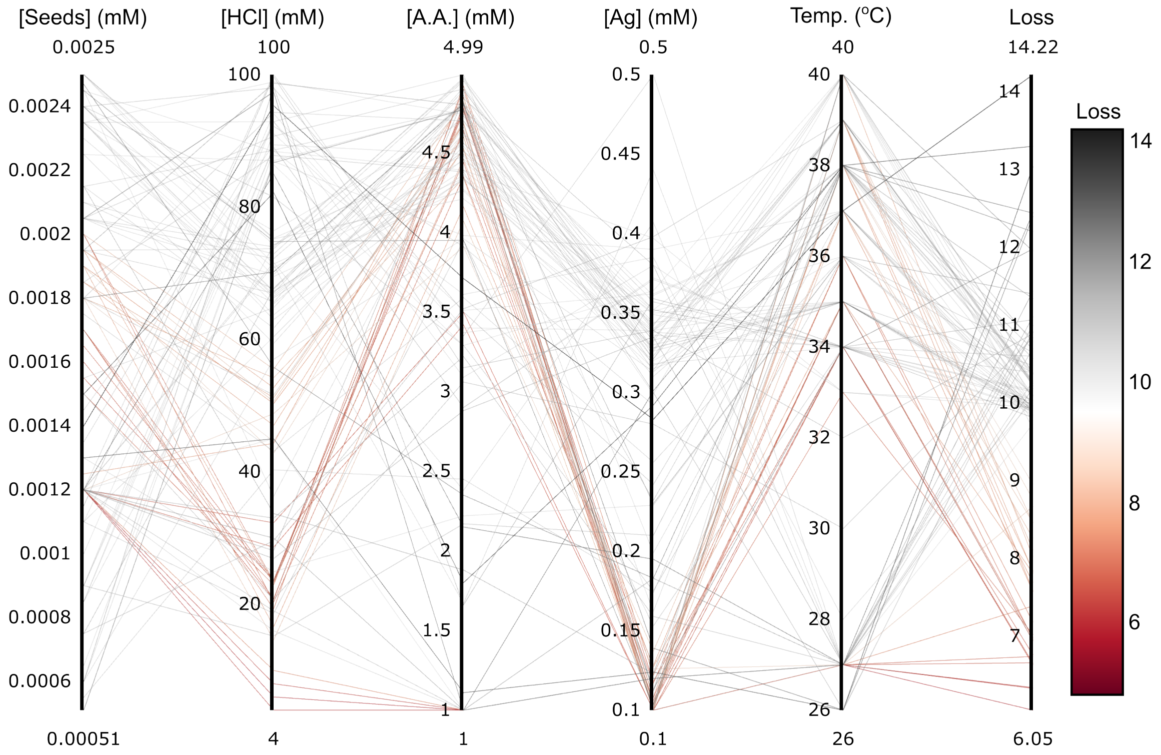

Figure S5: Parallel coordinate graph showing the different experimental conditions that resulted in the growth of similar quality AuNRs. Here, similar values of loss are referred to as similar quality of AuNRs.

Table S1: Table summarizing reaction conditions where AuNRs with similar UV-Vis-NIR response could be viably grown. All the volumes are given in μL.

**Vol. (Au seeds)**	**Vol. (1M HCl)**	**Vol. (100mM AA)**	**Vol. (10mM Ag^+)**	**Temp (°C)**	**Loss**
24.0	323	350	100	27	6.66
24.0	286	342	100	27	6.74
38.0	166	416	128	37	7.69
40.0	203	435	117	36	6.96
39.0	232	446	127	35	7.81
40.0	234	444	106	35	6.98
34.0	208	476	108	35	6.94
25.0	443	485	108	38	7.57
34.0	193	492	122	36	7.18
33.0	234	478	104	35	6.94
34.0	236	477	118	34	6.76
32.0	207	480	104	34	6.63
31.0	236	464	100	34	6.65
30.0	229	490	100	33	7.02
37.0	470	468	104	38	7.84
38.0	503	452	100	39	7.58
39.0	527	460	100	39	7.83
24.0	40	100	120	27	6.33
24.0	60	100	120	27	6.05
24.0	80	100	120	27	6.34
24.0	100	100	120	27	7.39

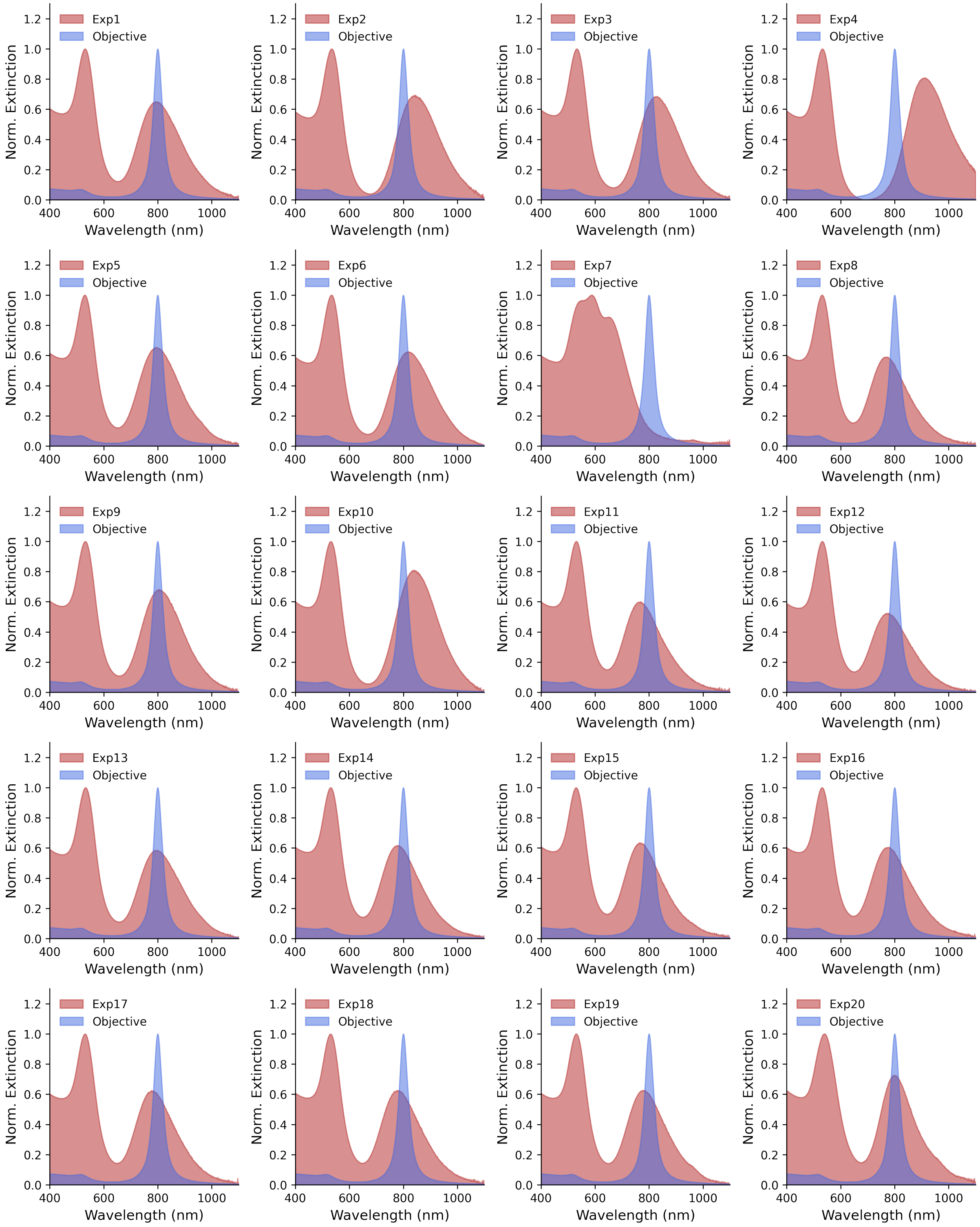

Figure S6: UV-Vis-NIR spectrum of all the experiments performed during the unsuccessful iteration towards optimization of AuNRs.

S9 UV-Vis-NIR for all Experiments in Figure 3

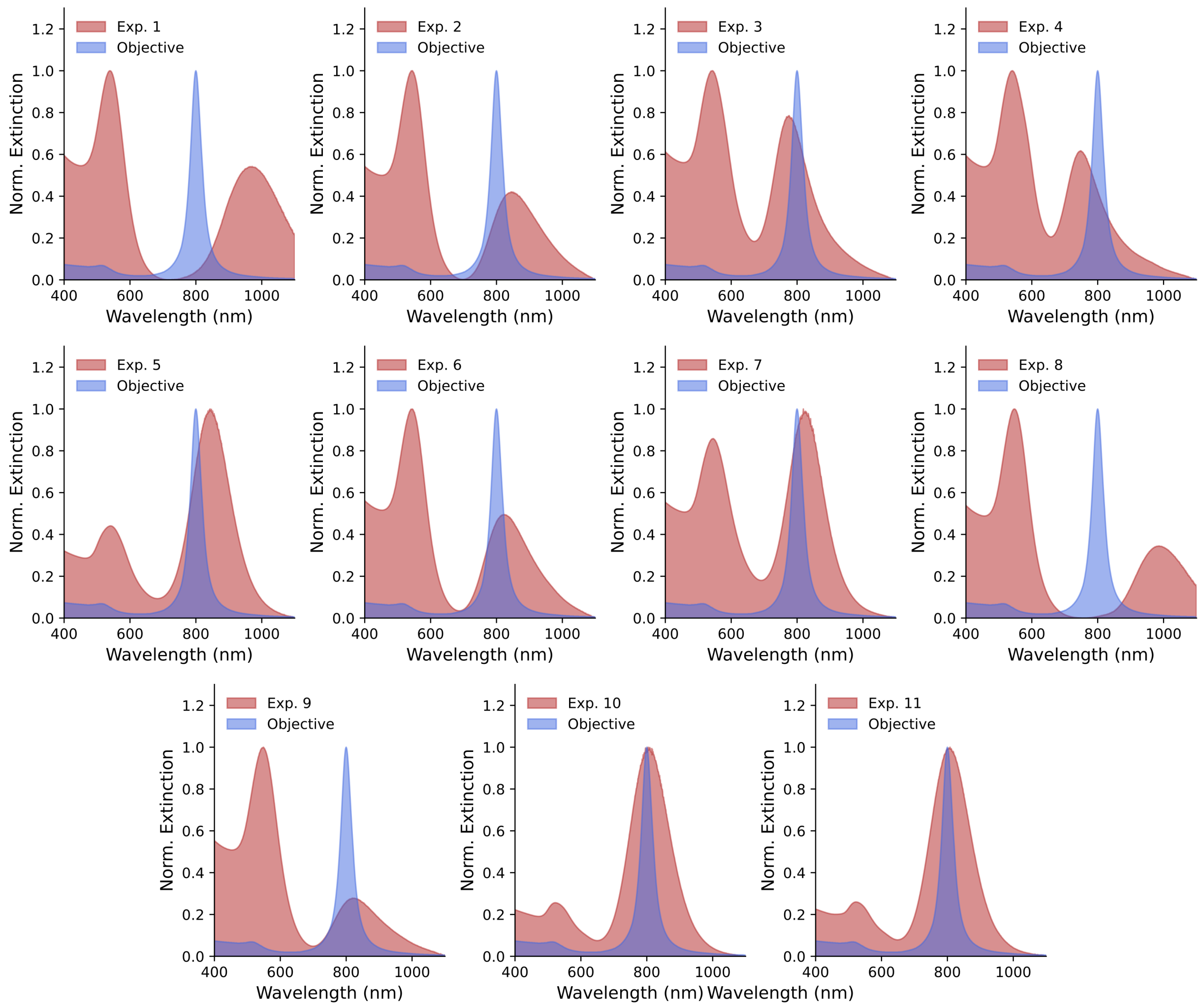

Figure S7: UV-Vis-NIR spectrum of all the experiments performed during the multi-objective optimization of AuNRs.

S10 Analysis of AuNR Growth Conditions

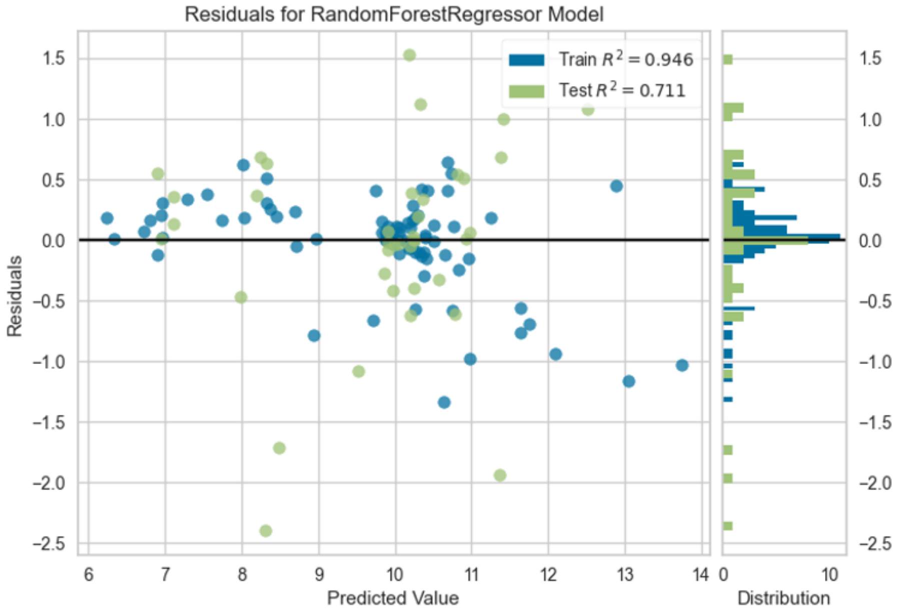

Figure S8: Figure demonstrating the residuals obtained for training and testing data.

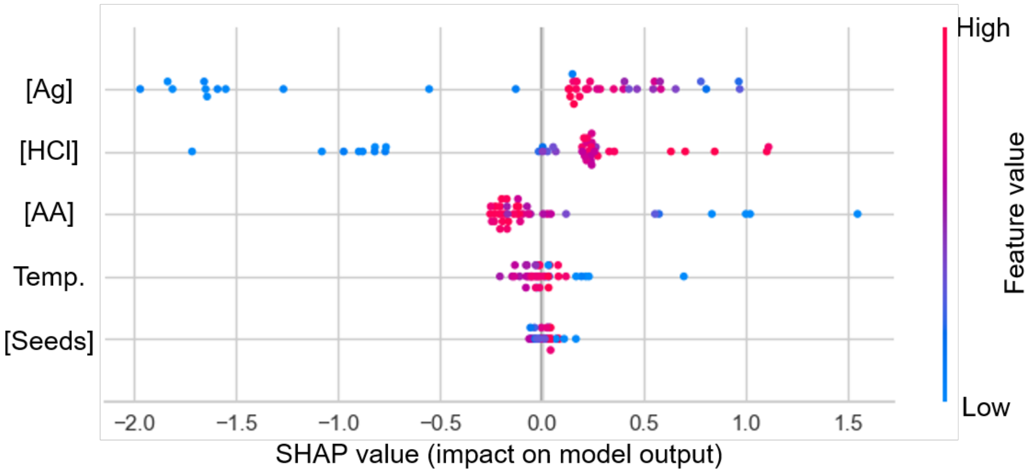

Figure S9: Shapley additive explanations (SHAP) analysis of the Random Forest Regressor model for the synthesis of AuNRs. The figure shows the impact of each reaction condition on loss.

S11 Variation in loss values in response to peak mismatch

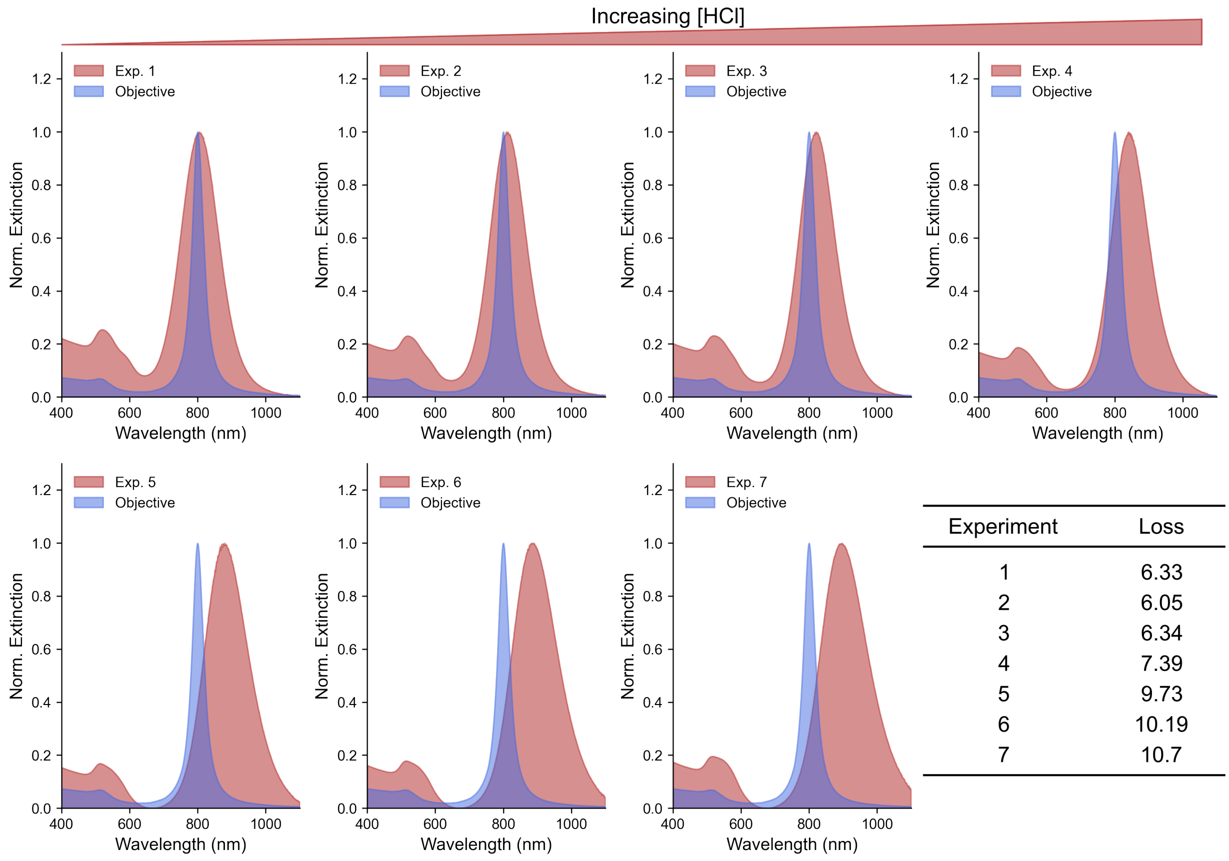

Experiment	Loss
1	6.33
2	6.05
3	6.34
4	7.39
5	9.73
6	10.19
7	10.7

Figure S10: UV-Vis-NIR spectrum of all the experiments performed by varying the concentrations of HCl during the synthesis of AuNRs. In Experiments 1-7, the volumes of 1M HCl were varied as follows: 40, 60, 80, 100, 120, 160, and 190 μL respectively.

S12 Parital dependence plots

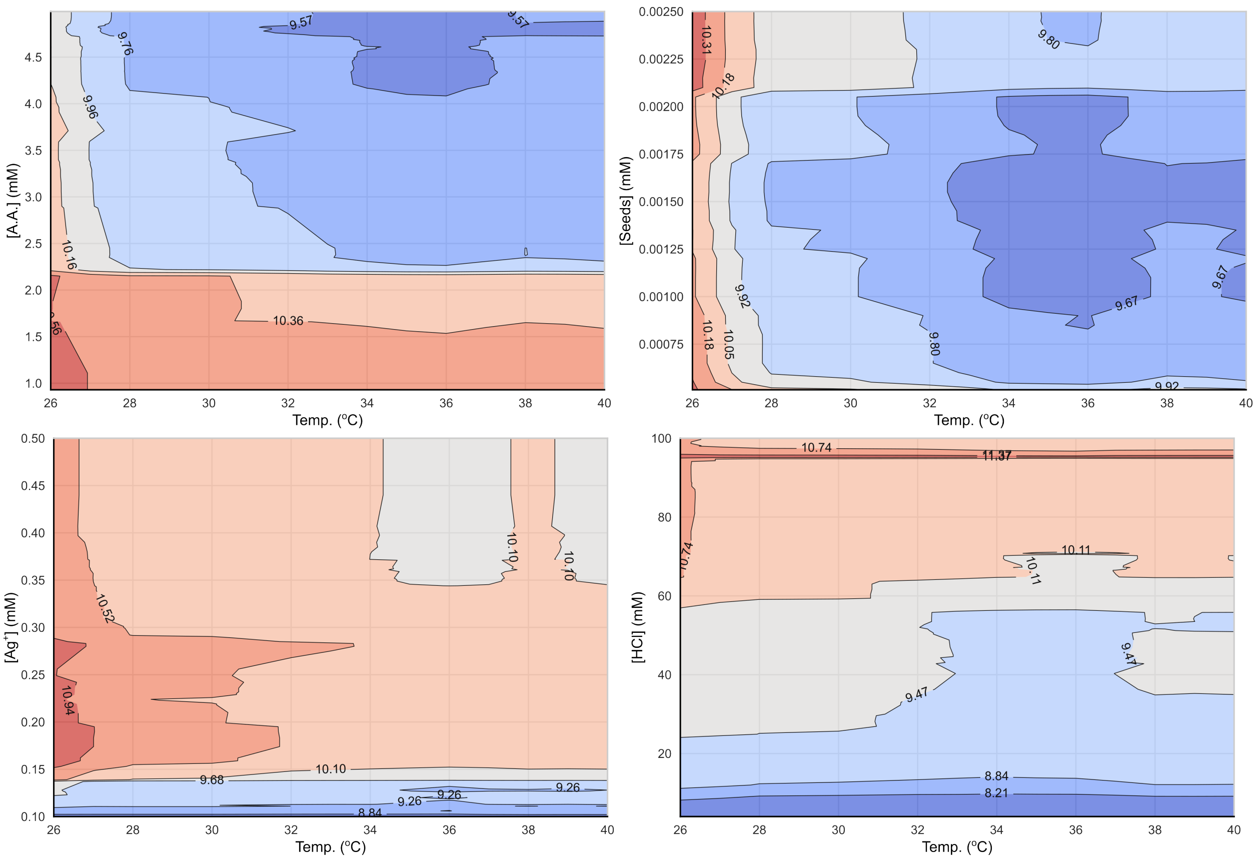

Figure S11: Partial dependence plots showing the collective effect of different reaction conditions on the predicted loss values.

S13 Conditions tested

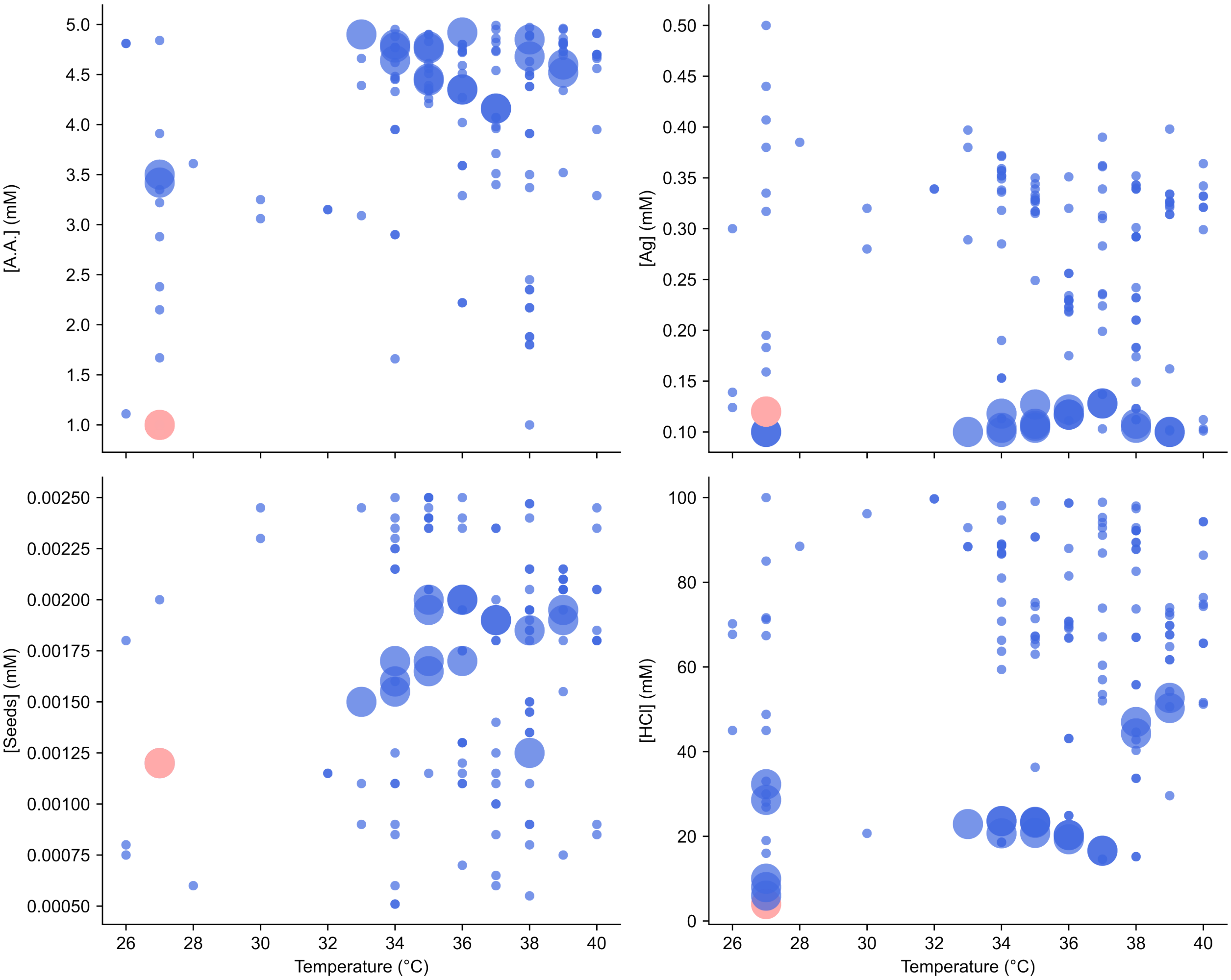

Figure S12: The collective effect between temperature and a) A.A., b) Ag^+, c) seeds, and d) HCl on the loss values of obtained AuNRs. Here, dots with large sizes indicate the formation of AuNRs with low loss values (loss less than 8). The pink circles denote the conditions used during El-Sayed synthesis.

References

(1) Myroshnychenko, V.; Rodríguez-Fernández, J.; Pastoriza-Santos, I.; Funston, A. M.; Novo, C.; Mulvaney, P.; Liz-Marzán, L. M.; De Abajo, F. J. G. Modelling the optical response of gold nanoparticles. *Chem. Soc. Rev.* **2008**, *37*, 1792–1805.

(2) De Abajo, F. G.; Howie, A. Retarded field calculation of electron energy loss in inhomogeneous dielectrics. *Phys. Rev. B* **2002**, *65*, 115418.

(3) Johnson, P. B.; Christy, R.-W. Optical constants of the noble metals. *Phys. Rev. B* **1972**, *6*, 4370.
